# Molecular Mechanisms Related to Hormone Inhibition Resistance in Prostate Cancer

**DOI:** 10.3390/cells8010043

**Published:** 2019-01-11

**Authors:** Veronica Mollica, Vincenzo Di Nunno, Alessia Cimadamore, Antonio Lopez-Beltran, Liang Cheng, Matteo Santoni, Marina Scarpelli, Rodolfo Montironi, Francesco Massari

**Affiliations:** 1 Division of Oncology, S. Orsola-Malpighi Hospital, 40138 Bologna, Italy; veronica.mollica7@gmail.com (V.M.); dinunnovincenzo88@gmail.com (V.D.N.); 2 Section of Pathological Anatomy, Polytechnic University of the Marche Region, School of Medicine, United Hospitals, 60126 Ancona, Italy; alessiacimadamore@gmail.com (A.C.); m.scarpelli@univpm.it (M.S.); 3 Department of Surgery, Cordoba University Medical School, 14071 Cordoba, Spain; em1lobea@gmail.com; 4 Department of Pathology and Laboratory Medicine, Indiana University School of Medicine, Indianapolis, IN 46202, USA; liang_cheng@yahoo.com; 5 Oncology Unit, Macerata Hospital, 62012 Macerata, Italy; mattymo@alice.it

**Keywords:** hormone inhibition resistance, prostate cancer (PCa), castration-resistance PCa, AR splice variants, epigenetic mechanisms

## Abstract

Management of metastatic or advanced prostate cancer has acquired several therapeutic approaches that have drastically changed the course of the disease. In particular due to the high sensitivity of prostate cancer cells to hormone depletion, several agents able to inhibit hormone production or binding to nuclear receptor have been evaluated and adopted in clinical practice. However, despite several hormonal treatments being available nowadays for the management of advanced or metastatic prostate cancer, the natural history of the disease leads inexorably to the development of resistance to hormone inhibition. Findings regarding the mechanisms that drive this process are of particular and increasing interest as these are potentially related to the identification of new targetable pathways and to the development of new drugs able to improve our patients’ clinical outcomes.

## 1. Introduction

Prostate Cancer (PCa) is the most common malignancy in men [1]. Early stages of the disease could be managed by different approaches, including non-invasive approaches (watchful waiting and active surveillance) or radical approaches represented by radiation therapy, surgery or brachytherapy [2,3,4,5,6,7,8]. Similarly to early stages, advanced stages of the disease could benefit from several approaches. However, androgen deprivation therapy (ADT) alone or in combination with other systemic treatments (abiraterone or docetaxel) in high-risk newly diagnosed metastatic patients, has been the cornerstone of advanced prostate cancer management [9,10,11,12,13,14]. Due to the high sensitivity of tumor cells to hormone deprivation therapy, the administration of ADT results in significant clinical benefit and can prolong the survival of patients. Nonetheless, after a variable time of ADT sensitivity (12–24 months), tumor cells could escape from hormone inhibition and lead to disease progression. This is due to the development of castration resistant prostate cancer (CRPC), which is defined as a prostate cancer that has progressed despite castrate levels of serum testosterone (<50 ng/dL). The acquisition of castration resistant status does not mean that tumors cells are insensible to hormone inhibition. New anti-androgens are able to improve hormone depletion or androgen receptor inhibition resulting in clinical benefit in this population as well. The administration of these agents in early phases of the disease, such as non-metastatic prostate cancer, has also demonstrated to improve biochemical progression of the disease [15,16,17,18,19,20,21,22]. Unfortunately, tumor cells develop different mechanisms that are able to overcome these hormonal pressures and become resistant to these treatments after a variable time of response. The identification of molecular and genetic mechanisms related to the development of castration resistant prostate cancer as well as the strategies adopted by tumors for escape from new anti-androgen agents are of particular interest, as new therapeutic strategies are strongly needed to improve patient’s outcomes [23].

One of the most important obstacles in this line of work is the availability of patients’ tumor tissue. As re-biopsy can be difficult, different approaches have been tested to acquire cells and monitor cells genomic expression modification during treatment. In vitro studies could not reflect the exact genomic modification observed in our patients. The research of circulating tumor cells or circulating tumor DNA represent a promising approach, even if to date only one device (CellSearch^®^) has been approved [24,25].

In this review, we wanted to summarize the current knowledge about cellular and genomic modification related to the acquisition of resistance to hormone inhibition; in addition, we wanted to focus our attention to possible strategies to overcome these mechanisms and ongoing clinical trials.

## 2. Androgen Receptor and Splice Variants

All prostate cells, both normal glandular epithelium and cancer cells, are strictly dependent on androgens, as they express the human androgen receptor (AR). The *AR* gene is mapped on chromosome Xp11-12 and belongs to the steroid hormone receptor genes family. The full-length AR (*AR-FL*) contains a N-terminal domain (NTD), encoded by exon 1 and that contributes in regulating the transcription of the *AR* gene, a DNA-binding domain (DBD), encoded by exon 2 and 3, a hinge region, and a ligand binding domain (LBD), encoded by exons 4–8 and which recognize the hormone with specificity and selectivity [26,27].

The AR is a ligand-dependent transcription factor activated by androgen hormones: in the absence of ligands (dihydrotestosterone and testosterone) it is located in the cell cytoplasm and translocates into the nucleus once the androgen hormone binds it. In the nucleus, the AR dimerizes creating a homodimer, binds through its DBD to androgen response elements (ARE) in the promoter regions of androgen response genes, recruits coregulatory proteins and epigenetic factors and stimulates downstream expression of genes that promote cell proliferation and survival [26,27].

The regulatory activity of the AR upon cellular survival makes its persistent activation a fundamental process at the basis of prostate cancer cells’ development and progression.

*AR’s* gene can undergo alternative splicing that generates constitutive active ARs independent from the presence of the ligand. AR splice variants (AR-Vs) can be characterized by the deletion of the LBD, which acts as a repressor for the receptor, or of the NTD, disrupting important regions that interacts with activation function 2 (AF2) in the LBD resulting in enhanced activation of AF2 and AR-mediated gene activation [28].

**Figure 1:**
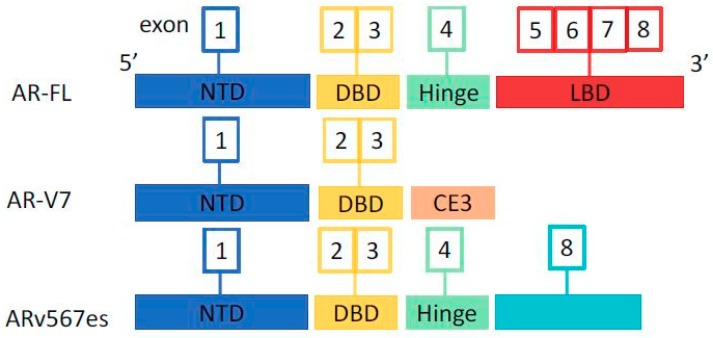
Androgen receptor (AR) and splice variants. AR-FL: AR full length. NTD: N-terminal domain. DBD: DNA-binding domain. LBD: ligand binding domain. CE3: cryptic exon 3.

Many *AR* splice variants have been identified in prostate cancer and have been associated with the development of resistance to ADT, the most widely studied and clinically meaningful being the AR-V7 (or AR3) and the ARv567es (or AR-V12). AR-FL and the main splice variants structures are represented in Figure 1.

ARv567es lacks the exons 5–7 that encode the LBD but retains the hinge region of exon 4 necessary for nuclear translocation, producing a constitutively active AR isoform with transcriptional activity independent from the ligand. This variant can be expressed in benign and malign prostate tissues, however, its levels increase during ADT creating a state of resistance to castration [26,29].

AR-V7 is a truncated isoform of the AR derived from aberrant mRNA splicing of AR exons 1, 2, 3, inclusion of the cryptic exon 3 (CE3) and loss of exons 4–8 that encode the LBD of the AR-FL. As a result, AR-V7 lacks the LBD and contains in its cryptic exon the NLS, allowing an intra-nuclear localization independent of the ligand and persistent activation of AR leading to an excess of survival signaling and growth of PCa cells. The independent activity of AR-V7 from the ligand is at the basis of the resistance to anti-androgen therapies and the development of castration resistant disease [26,27,30].

AR-V7 has been associated with poor prognosis in PCa metastatic to the bones [31], increased risk of biochemical disease recurrence after radical prostatectomy [32], and progression supporting epithelial-to-mesenchymal transition that contributes to metastatic spreading [33]. Furthermore, the main area of interest regarding AR-V7 is its involvement in developing resistance to anti-androgen treatments leading to a state of castration resistance, as explained later.

## 3. Mechanisms of Resistance to Therapies

AR alterations have been associated not only with the development of the state of castration resistance but also to resistance to abiraterone and enzalutamide. Many genetic and epigenetic mechanisms lie beneath this state of resistance. Resistance to AR-targeted therapies in CRPC can be attributed to various mechanisms corresponding to different histological, clinical, and molecular profiles: restored AR signaling, AR bypass signaling, and complete AR independence [34].

Restored AR signaling can be due to molecular alterations like AR-activating mutations, AR active splice variants, and intratumoral dihydrotestosterone synthesis from adrenal precursors.

In a minority of patients with CRPC, AR activating mutations in the LBD have been found through genomic sequencing studies, the principal four point mutations being L702H, W742C, H875Y, and T878A [35]. AR carrying T878A and H875Y mutations are activated, rather than inhibited, by the anti-androgens nilutamide and flutamide, resulting in transcriptional induction of AR target genes during anti-androgen therapy [36]. Similarly, W742C mutations resulted in enhanced AR transcriptional activity driven by bicalutamide [34,37]. H875Y, T878A, and L720H (not linked to anti-androgen resistance) are more frequent that W742C, even though bicalutamide therapy is the most currently used in clinical practice. The explanation proposed is that AR with these mutations can be promiscuously activated by other steroid ligands (adrenal androgens, oestrogen, and progesterone (or in the case of L702H, by glucocorticoids), thus leading to disease progression [34,38,39].

AR splice variants have been implicated in developing of acquired resistance to ADT, abiraterone, and enzalutamide. The structural alterations of AR-Vs confer androgen-independent activity to the AR isoforms that is correlated with nuclear localization independently by the presence of the ligand. AR-V567es and murine mAR-V4 maintain exons 3 and 4 that are responsible for the complete nuclear localization signal, while the other isoforms are truncated after exon 3 and are mostly cytoplasmic. AR-V7 is an exception, because it has constitutive nuclear localization and transcriptional activity without a full nuclear localization signal [26]. The selective pressure of androgen deprivation therapies seems to select AR-V7 expressing clones resistant to these therapies: as a result, AR-V7 expression appears higher in CRPC than in hormone naïve PCa and is enhanced after enzalutamide or abiraterone therapy [30]. Prostate cancer cells and CRPC xenografts under enzalutamide or abiraterone present a high expression of LBD-truncated AR-V7 and AR-V567es that produce an overexpression of UBE2C gene leading to treatment resistance [40].

In preclinical studies, knockdown of these variants conferred sensitivity to enzalutamide, underlining their role in the development of resistance to enzalutamide by persistently activating the AR-transcriptional program [41]. Furthermore, the finding that AR-V7 protein expression between different metastases in the same patient is heterogeneous could indicate that the resistance to endocrine therapies could rely on multiple mechanisms, suggesting that the therapeutic approach should be multiple as well [30]. The increasing knowledge that AR-V7 expression could be a mechanism of developing castration resistance is underling the importance of targeting AR-V7 with novel therapeutic strategies when the tumor is still sensitive to ADT in order to maintain a longer state of castration sensitivity and improve patient’s outcome.

Another mechanism of resistance to androgen receptor inhibitors can be bypassing AR signaling by activating the downstream hormone receptor pathway through a different hormone receptor like glucocorticoid receptor, thus overcoming AR dependency [34]. Furthermore, resistance to anti-androgen therapies can be due to a state of complete androgen receptor independence. It is known that tumor molecular heterogeneity can lead to a mixture of cells that express different levels of AR. In addition, after enzalutamide or abiraterone treatment, the tumor can progress with more aggressive variants characterized by a reduced or absent AR expression and histological features of neuroendocrine differentiation. This latter type of PCa express molecular alterations like loss of *RB1*, *PTEN* and *TP53* mutations, amplification of *MYCN* and Aurora kinase A [34].

## 4. Epigenetic Mechanisms

Genetic and epigenetic alteration contribute to the clinical behavior of prostate cancer, which ranges from indolent tumors to more aggressive ones. Epigenetic changes include DNA methylation and histone acetylation and deacetylation, which can occur in various genes and may represent a potential therapeutic target.

DNA methylation mechanisms associated with prostate cancer development, and progression are: DNA hypermethylation of CpG sites in gene-promoter regions due to DNA-methyltransferases that lead to transcriptional silencing and DNA methylation in the gene body [42].

Many genes have been implicated in DNA methylation changes correlated to prostate carcinogenesis and tumor progression. Among them, hypermethylation of the AR promoter and the consequent loss of AR expression was described in up to 30% of CRPCs [43].

Geybels et al. [44] investigated the differences in DNA methylation between malign and benign prostate tissue and identified three genes (*SCGB3A1, HIF3A, AOX1*) differently methylated between the two tissues associated with promoter methylation and reduced gene expression.

Furthermore, a study of epigenome-wide DNA methylation profiling by the same authors found different methylated CpGs in five genes (*PI15, ALKBH5, ATP11A, FHAD1, KLHL8*) and three intergenic regions that differ in metastatic lethal prostate cancers and non-recurrent ones [45]. Another study found an association of CpGs sites hypermethylation of *FLNC, EFS, ECRG4, PITX2, PDLIM4*, and *KCNMA1* genes and local and systemic recurrence [46].

DNA-methyltransferase (DNMTs) are a class of enzymes that work in association with the apoptosis-modulating protein DAXX. The latter is a transcription repressor that promotes chromosome stability: it has been found overexpressed in PCa and its expression level has been correlated with the Gleason score and cancer development [47]. DAXX targets DNMT1 onto specific sites in the genome resulting in their hypermethylation and epigenetic silencing [48].

Histone modifications are another epigenetic mechanism controlling gene expression correlated with development and progression of PCa and consist of posttranslational changes in the N-terminal tails of histones: acetylation/deacetylation, methylation, phosphorylation, deamination, citrullination, ubiquitination, and ADP ribosylation [49]. The enzymes that regulate the acetylation status of histones are histone acetyltransferases (HATs) and histone deacetylases (HDACs). The acetylation of histones mediated by HATs result in an open chromatin structure that makes DNA more accessible by transcription factors. HATs can be distinguished into type A, located in the nucleus, and type B, located in the cytoplasm. Some type A HATs have been associated with proliferation and disease progression in PCa, like p300, PCAF, and Tip60 [42]. Acetylation of histone H3 near the AR-bound chromatin has been shown to reduce androgen dependence in CRPC models [50]. On the other hand, deacetylation of core histones in the N-terminal regions results in lower DNA accessibility and in gene silencing. HDAC 1 and 3 are involved in modulation and transcription of AR and the inhibition of these enzyme resulted in decreased AR synthesis [42]. HDACs can deacetylate the AR, thus inhibiting its activity with various mechanisms like regulation of heat-shock protein 90, a chaperone that controls AR nuclear localization and activation through its acetylation or deacetylation [51]. Furthermore, HDAC sirtuin (SIRT) 1 can regulate cellular growth through AR deacetylation [52], while downregulation of SIRT2 deacetylase can lead to acetylation of histone H3K18, which has been associated with poorer outcome and decreased sensitivity to ADT [43,53].

The enhancer of zeste homolog 2 (EZH2) is a histone methyltransferase enzyme member of the Polycomb Repressive Complex 2 (PRC2) [54]. EZH2 represses lineage specifying factors, thus favoring metastatic progression by promoting stemness and epithelial-mesenchymal transition [43]. EZH2 controls several transcriptional activities and its increased expression is associated with silencing of different genes, including for example p53 [55]. Increased expression of this enzyme has been associated to worse clinical features and AR resistance. Furthermore, the concurrent high expression of EZH2 and TOP2A can identify a subgroup of aggressive PCa more likely to progress to metastatic disease [56,57]. Wang et al. [58] recently correlated the histone lysine demethylase/dioxygenase KDM8/JMJD5 expression to development of castration resistant status. This appears mainly mediated by an interaction carried out by EZH2, which is directly targeted by KDM8. Thus, by inhibiting EZH2, it was possible restore sensitivity to hormone therapy [58]. Furthermore, there is an increasing amount of evidence that ADT can lead to promotion of neuroendocrine differentiation by activation of cAMP response element-binding protein (CREB), though this mechanism is still largely unknown. CREB lead to a state of castration resistance because its binding protein, a histone acetyltransferase, act as an AR coactivator in transcriptional activation of AR target genes. It has been shown that ADT can activate CREB, which enhances EZH2 activity that consequently upregulates neuroendocrine markers and downregulates trombospondin-1, an anti-angiogenetic factor, thus promoting angiogenesis, a characteristic of neuroendocrine tumors that appears to be highly vascularized [59,60].

Acknowledging the epigenetic mechanisms involved in PCa is important for their potential use as targets for novel therapeutic approaches.

## 5. How to Overcome Resistance to AR Inhibition

**Figure 2:**
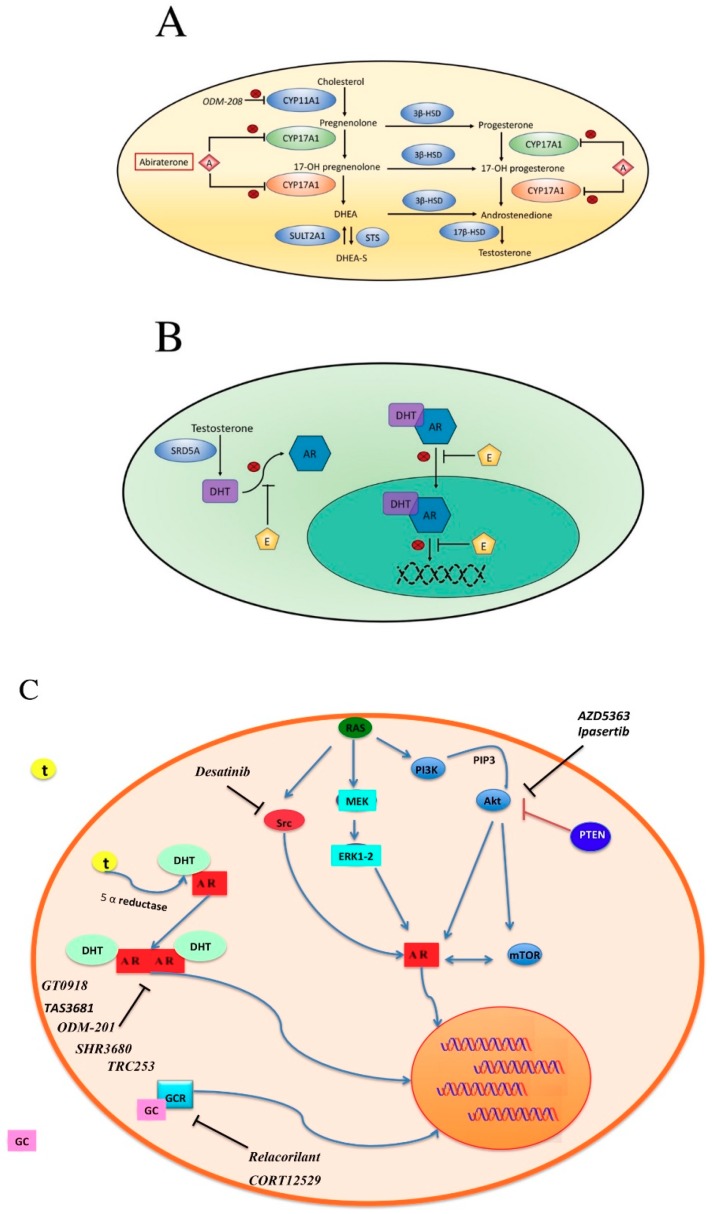

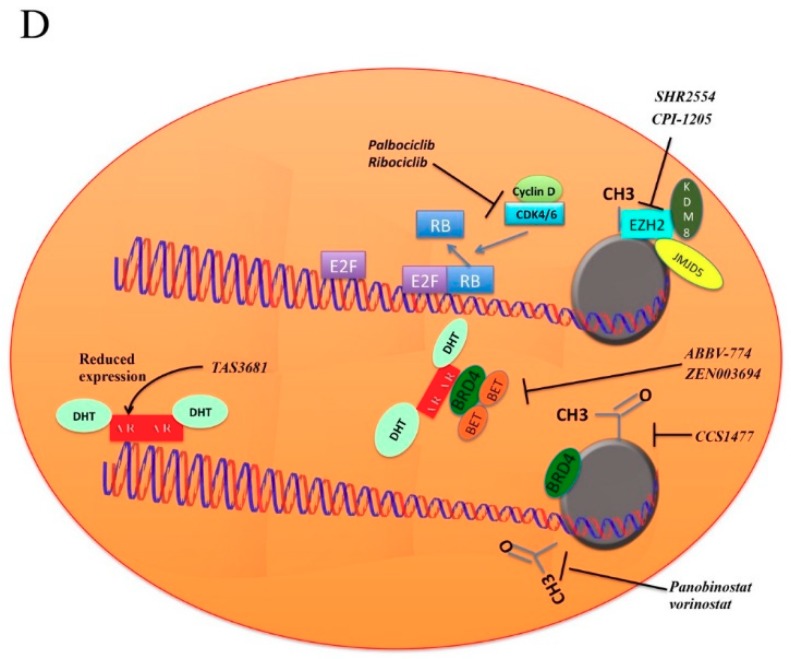
Androgenesis inhibition. (**A**) Adrenal androgen steroidogenesis. CYP17A1: cytochrome P450 family 17 subfamily A polypeptide 1 with two activities: 17α-hydroxylation (green) and 17, 20-lyase (orange); both activities are inhibited by abiraterone. CYP11A1: cytochrome P450 family 11 subfamily A polypeptide 1. 3β-HSD: 3β-hydroxysteroid dehydrogenase. 17β-HSD: 17β-hydroxysteroid dehydrogenase. SULT2A1: bile salt sulfotransferase. STS: steryl-sulfatase. DHEA: Dehydroepiandrosterone. DHEA-S: Dehydroepiandrosterone sulfate. A: abiraterone; (**B**) mechanism of action of Enzalutamide. Enzalutamide inhibits AR in several ways: competing with DHT for binding, inhibiting nuclear translocation, blocking DNA, and co-factor binding. E: enzalutamide. AR: androgen receptor. DHT: dihydrotestosterone. SRD5A: steroid 5α-reductase; (**C**,**D**) proposed mechanisms related to hormone inhibition resistance and experimental inhibitors under evaluation in clinical trials. Akt: protein kinase B; AR: Androgen Receptor; Src: proto-oncogene tyrosine-protein kinase Src; Ras: Ras GTPase family proteins; MEK: Mitogen-activated protein kinase; ERK1-2: Extracellular signal-regulated kinases; PI3K: Phosphoinositide 3-kinase; PTEN: Phosphatase and tensin homolog; mTOR: mammalian target of rapamycin; GCR: glucocorticoid receptor; GC: Glucocorticoid; T: testosterone; DHT: Dihydrotestosterone; KDM8: Lysine demethylase 8; JMJD5: JmjC domain containing protein 5; EZH2: enhancer of zeste homolog 2; BRD4: bromodomain containing protein 4; BET: Bromodomain and extra terminal family proteins; RB: retinoblastoma protein; E2F: transcription factors E2.

As already mentioned, one of the most important hormone-inhibition-escape-strategy adopted by prostate cancer cells is the alternative splicing of the AR which results in the expression of different alternative AR-Vs. In brief, there are two approaches which could be adopted to overcome this alternative splicing: one consists on the adoption of agents able to modulate AR-Vs transcription, while the other is the development of drugs able to target these specific alternative variants (Figure 2).

Regarding the first approach, several strategies targeting different pathways are currently under investigation.

### 5.1. BET Pathway

The bromodomain and extra-terminal (BET) family proteins and the bromodomain-containing proteins (BRD) are able to recognize and bind acetylated lysine residues in histones and other proteins through their bromodomains (BDs), modulating recruitment of other proteins and regulating DNA transcription (Figure 2D). Different BRD are able to interact with AR in nucleus, the more common association being the BRD4-AR interaction. As further evidences seem to show a key role of this protein as mediator of AR and AR-Vs transcription, the inhibition of BET pathways is an attractive option to overcome resistance to androgen inhibition [61,62]. To date, a phase I/II clinical trial is currently evaluating safety and clinical activity of the combination between Enzalutamide and the BET inhibitor ZEN003694 (NCT02711956, Figure 2D) in patients with mCRPC while two phase I studies are testing safety profile of ABBV-774 alone (NCT03360006) or in combination with the Bcl-2 inhibitor Venetoclax (NCT02391480, Figure 2D) in solid and hematological tumors.

### 5.2. Cyclins

Increasing interest in the development of cyclin inhibitors is mainly due to the important results obtained in metastatic breast cancer. Cyclins are a heterogeneous group of proteins which acts in different point of cell cycle. Thus, we recognized four types of cyclin according to the different phase of cell division: G_1_/S cyclins, S cyclins, M cyclins and G_1_ cyclins. In prostate cancer, there are evidences that seem to correlate cyclins expression to AR expression, radio-resistance and hormone inhibition resistance [63,64,65]. Of interest, Pal et al. performed an evaluation on circulating tumor cells (CTCs) of patients with prostate cancer who received abiraterone or enzalutamide [65]. They evaluated different genetic expression of CTCs in patients with sensitivity or acquired resistance to treatment. Of the several potential genes correlated to acquired resistance, an up-regulation of cyclin D1 in drug resistant CTCs was observed. Furthermore, when they induced cyclin hyper-expression in tumor cell tumor culture they observed that cells were refractory to enzalutamide production. This resistance was partially avoided through the adoption of cyclin-dependent kinase inhibitors. Not surprisingly, the adoption of cyclin-dependent kinase inhibitors is a promising strategy under investigation alone (palbociclib, NCT02905318, Figure 2D) or in combination with enzalutamide (ribociclib, NCT02555189, Figure 2D) or docetaxel (ribociclib, NCT02494921).

### 5.3. Histone Deacetylase Inhibitors

The non-selective histone deacetylase inhibitor panobinostat (Figure 2D) and the suberolylanilide hydroxaminic acid vorinostat (Figure 2D) have been evaluated in association with docetaxel in patients with advanced solid malignancies (vorinostat) and metastatic CRPC (panobinostat) [66,67,68,69]. Unfortunately, the combination of vorinostat and docetaxel resulted in excessive dose-limiting toxicity [66] while a better safety profile has been observed with the combination of panobinostat and docetaxel [67]. More recently, a phase II study evaluating intravenous panobinostat failed to show significant clinical activity of this drug [68]. Despite these results, a study carried out by Ferrari et al. on xenograft and isogenic cell line demonstrated that the addiction of panobinostat to bicalutamide prolonged tumor sensitivity to bicalutamide, suggesting an adoption of this drug in combination with androgen deprivation therapy [69]. Of note, the combination between an acetylase inhibitor (CCS1477) and abiraterone is under evaluation in a phase I/II trial (NCT03568656).

### 5.4. EZH2

As already discussed, inhibition of the histone methyltransferase EZH2 could restore sensitivity to hormone therapy. Currently, there are two ongoing phase I/II studies evaluating the EZH2 inhibitor CPI-1205 (Figure 2D) in association with Abiraterone and enzalutamide (NCT03480646) as well as the combination between the androgen receptor antagonist SHR3680 (Figure 2C) and the EZH2 inhibitor SHR2554 (NCT03741712, Figure 2D).

Other approaches have been considered to overcome acquired resistance to hormonal inhibition. As already described, specific agents able to target altered splicing ARVs are under evaluation as well as drugs with higher antagonistic power against AR. However, several other altered pathways seem to be implicated in the development of AR inhibition resistance; thus, the adoption of combined treatment between these pathways and AR could be a winning strategy.

### 5.5. PI3K-AKT-mTOR

Through the regulation of an extraordinary number of effectors able to modulate phosphorylation, transcription or translation of several target points the phosphoinositide-3-kinase (PI3K)-AKT-mammalian target of rapamycin (mTOR) pathway modulate cellular process able to promote protein synthesis, proliferation, survival, differentiation, and metabolism. Not surprising, due to the large number of functions regulated by this pathway alteration in PI3K-AKT-mTOR are quite frequent in several tumor types [70]. About prostate cancer, deregulation of PI3K-AKT-mTOR signaling could be observed in 42 up to 100% of cases in localized and advanced disease, respectively [71]. Regarding the interaction between AR and PI3K-AKT-mTOR signaling, some in vivo studies have demonstrated that AR inhibition in mouse with PTEN loss could enhance PI3K-AKT-mTOR signaling mainly through the down-regulation of PHLPP, which is a negative regulator. PTEN is a tumor suppressor gene downregulated in approximately 50% of CRPC and its loss, leading to activation of PI3K-AKT-mTOR pathway, is associated with the development of castration resistance and poor outcomes [56,72]. AKT enhanced activity reduces AR dependence of prostate cancer cell; thus, it contributes to the development of castration resistant status [73,74,75]. Of interest, inhibition of PI3K-AKT-mTOR pathways leads to more aggressive tumors with acquired neuroendocrine features through the degradation of RE-1 silencing transcription factor (REST) protein [76]. Furthermore, the concomitant inhibition of AKT pathway and AR signaling seems to be correlated to an enhanced REST depletion and consequent promotion of differentiation toward a neuroendocrine subtype [76]. Currently, there are some AKT inhibitors under evaluation as mono-treatment (AZD5363, NCT02121639, Figure 2C) or in association with abiraterone (Ipasertib, NCT03072238, Figure 2C), which should be carefully considered taking into account the data suggesting the interaction between these two pathways in enhancing neuroendocrine differentiation.

### 5.6. SRC

The SRC gene encodes the proto-oncogene tyrosine protein kinase Src which plays an important role during embryogenic development and cell-growth. Evidences about its correlation with Androgen Receptor pathways and development of castration resistant status are further increasing. Szafran et al. performed an AR high content analysis to characterize the effects of specific compounds including Akt, Abl, and Src inhibitor on AR-V7 expression demonstrating that all these drugs were able to reduce the expression of this splice variant [77]. Moreover, Src seems to be able to promote translation to CRPC through sensitization of AR to intracrine androgen levels by the development of canonical and non-canonical Androgen receptor binding site associated genes [78]. This has led to the development of a combined strategy between the Src inhibitor dasatinib (Figure 2C), enzalutamide, and MEK inhibitor (NCT01990196) currently under investigation.

### 5.7. Other Strategies to Overcome Hormone Inhibition Resistance

Several compounds able to directly inhibit the AR are under investigation. Of these, TAS3681 (Figure 2C,D) is an oral AR antagonist able also to down-regulate AR. Additionally, this drug is able to inhibit the AR-V7 splice variants and so it seems to be active also in patients refractory to new anti-androgen inhibition [79]. Thus, a phase I study is evaluating its safety in patients with metastatic advanced prostate cancer (NCT02566772).

Other AR inhibitor and antagonist with higher AR affinity are also under investigation in different setting of the disease, alone or in association with other compounds (NCT02711956, NCT02972060, NCT02691975, NCT02826772, NCT02987829, NCT02933801, NCT03314324). ODM-208 (Figure 2A) is a small molecule able to target the CYP11A1 which is a key enzyme able to catalyze conversion from cholesterol to pregnenolone. Compared to Abiraterone which is a CYP17 inhibitor, it acts in an earlier step of androgen and steroid hormones development, preventing the synthesis of androstenedione, testosterone and also, deoxycorticosterone, and aldosterone. A phase I/II study is currently testing this drug and evaluating its safety (NCT03436485).

Another approach is represented by the administration of drugs able to antagonise the glucocorticoid receptor and so acting as antiglucocorticoid. Relacorilant (Figure 2C), an antiglucocorticoid conceived for patients with Cushing syndrome or alcoholism, is currently under evaluation in a phase I trial in combination with enzalutamide (NCT03674814), while the association between enzalutamide and the glucocorticoid receptor antagonist CORT12529 (Figure 2C) is under evaluation in a phase I/II trial (NCT03437941).

## 6. Conclusions

Management of patients progressed to standard androgen deprivation therapy have included agents able to improve hormone inhibition and are also active in castration-resistant prostate cancer. It is probable that combination strategies and development of new drugs will extend the benefit of these drugs and improve survival and clinical benefit of our patients. This goal is possible thanks to the increasing knowledge about genetic mechanisms related to acquisition of castration resistant status and this highlights the need of genomic assessment of tumor cells in different times of the disease. In this line of thought, a parallel evolution that should be considered is the adoption of techniques able to assess circulating tumor cells and DNA more and more effectively.

## Abbreviations

PCa	Prostate Cancer
ADT	Androgen Deprivation Therapy
CRPC	Castration Resistant Prostate Cancer
AR	Androgen Receptor
AR-FL	Full-Length AR
NTD	N-Terminal Domain
DBD	DNA-Binding Domain
LBD	Ligand Binding Domain
AR-Vs	AR splice Variants
DNMTs	DNA-methyltransferases
HATs	Histone Acetyltransferases
HDACs	Histone Deacetylases
CTCs	Circulating Tumor Cells
CYP17A1	Cytochrome P450 family 17 subfamily A polypeptide 1
CYP11A1	Cytochrome P450 family 11 subfamily A polypeptide 1
DHEA	Dehydroepiandrosterone
DHEA-S	Dehydroepiandrosterone sulfate
DHT	Dihydrotestosterone
Akt	Protein kinase B
PI3K	Phosphoinositide 3-kinase
PTEN	Phosphatase and tensin homolog
mTOR	Mammalian target of rapamycin
EZH2	Enhancer of zeste homolog 2
BRD4	Bromodomain containing protein 4
BET	Bromodomain and extra terminal family proteins
